# Publishing Trends in the Field of School-Based Health From 2014 to 2024: A Bibliometric Analysis

**DOI:** 10.7759/cureus.95951

**Published:** 2025-11-02

**Authors:** Christina Hermann, Robert P Olympia

**Affiliations:** 1 Pediatrics, Penn State Health Milton S. Hershey Medical Center, Hershey, USA; 2 Emergency Medicine and Pediatrics, Penn State Health Milton S. Hershey Medical Center, Hershey, USA

**Keywords:** bibliometric analysis, publishing trends, school-based health, school health services, school mental health services

## Abstract

School-based health interventions have been shown to significantly improve pediatric health outcomes. This study aims to analyze trends in research published on school-based health between 2014 and 2024. A comprehensive literature search was performed on PubMed. Data points abstracted from each article included publication details, study details, and research study topics.

A total of 953 publications were analyzed. A peak in annual publications was apparent in 2018-2019, followed by a decline. Nine- to 13-year-olds were the most studied population (50.9%). The most commonly assigned research topics were physical activity and nutrition (36%), health promotion (25%), mental health (19%), obesity (15%), and substance use (12%).

Research in the field of school-based health has evolved, but the overall annual number of publications is declining. Still, published data reveals that most school-based health studies have positive outcomes on the health of our pediatric population. These results underscore the need for continued research, particularly in underrepresented areas such as school-based health centers, telemedicine, minority youth, students with disabilities, and interventions targeting reproductive health, violence prevention, and socioeconomic disparities.

## Introduction and background

Background

Globally, school attendance among children has steadily increased since 2000 [1]. Today, over 90% of children of primary school age and over 80% of children of lower secondary school age attend school [2]. Given that children spend about one-third of their day in school, second only to time spent sleeping, schools are uniquely positioned to influence children’s long-term health and well-being [3].

Research has shown that health habits form early in life. A 2014 study at Brown University indicated that routines and habits are likely established by the age of nine and remain consistent at least through high school [4]. This makes the school years a critical period for fostering healthy behaviors and promoting lifelong well-being. Schools, therefore, have the unique opportunity to serve not only as learning environments, but also as centers for holistic health promotion.

School-based health research is an interdisciplinary field that spans public health, education, psychology, and the social sciences. It addresses a wide array of topics including nutrition, physical activity, mental health, substance use, hygiene, reproductive health, and social determinants of health.

The World Health Organization (WHO) and UNESCO recognized the potential of schools to promote health and launched the Health Promoting Schools initiative in 1992. This program encourages schools to foster environments that support both physical and mental well-being [2]. In 2021, the initiative was expanded to include a global resource package including standards, implementation guidance, and case studies, reaching an estimated 1.9 billion school-aged children and adolescents worldwide [5].

In the United States, school-based health centers (SBHCs), clinics located within or near schools, have become a crucial part of the public health infrastructure. These centers provide a range of services, including preventive care, mental health support, reproductive health services, and management of chronic conditions. SBHCs play an especially important role in improving healthcare access for underserved populations [6].

Rationale

Although a growing body of literature supports the effectiveness of school-based health interventions, demonstrating improvements in immunization rates, physical activity, nutrition, mental health, and substance use, there is limited understanding of how this field has evolved over time [7-9]. Existing research focus on specific topics or outcomes but have not provided a comprehensive assessment of broader publication trends, research focus, or thematic gaps.

Bibliometric analysis, a quantitative method for evaluating patterns in scientific output and thematic focus, offers a valuable lens to examine the scholarly landscape. While prior bibliometric studies have explored related pediatric topics, including childhood obesity, and adolescent mental health [10,11], none have specifically examined school-based health despite its growing relevance and multidisciplinary nature.

Objective

This bibliometric study aims to characterize the evolution of school-based health research over the past decade (2014-2024). Specifically, we sought to (i) analyze trends in publication volume over time; (ii) identify common research topics, populations, study designs, and journal types; and (iii) highlight underrepresented areas to inform future research priorities.

By providing an overview of trends and gaps, we aim to support interdisciplinary collaboration and help guide future research efforts to strengthen the role of schools in promoting child and adolescent health.

## Review

Methods

Search Strategy

A bibliometric analysis was conducted using a structured literature search performed exclusively in PubMed in July 2024. PubMed was selected due to its comprehensive coverage of clinical and biomedical literature and its standardized, reproducible filter options.

The search targeted school-based health research published between January 1, 2014 and July 1, 2024. A combination of title/abstract keywords and Medical Subject Headings (MeSH) terms was used to identify relevant studies. The following search string was applied: "school-based health"[Title/Abstract] OR "School Health Services"[MeSH Terms] OR "School Mental Health Services"[MeSH Terms] AND (y_10[Filter]) AND (clinicalstudy[Filter]) AND (english[Filter]) AND (child[Filter] OR adolescent[Filter])

The following filters were applied simultaneously: (i) Article type: clinical study; (ii) Language: English; (iii) Age groups: Child (6-12 years) and Adolescent (13-18 years); (iv) Publication date range: 2014-2024.

Eligibility Criteria

The inclusion criteria were as follows: (i) Published between 2014 and 2024; (ii) written in English; (iii) involved school-aged children or adolescents (ages six to 18 years); and (iv) classified as clinical studies by PubMed.

The exclusion criteria were the following: (i) Non-English language publications; (ii) review articles, commentaries, or editorials; (iii) studies focused exclusively on college-aged or preschool populations; and (iv) conference abstracts or grey literature.

Screening and Selection Process

All screening and data abstraction were conducted by a single reviewer. This included title and abstract screening, full-text review, inclusion/exclusion decisions, and data extraction.

**Figure 1:**
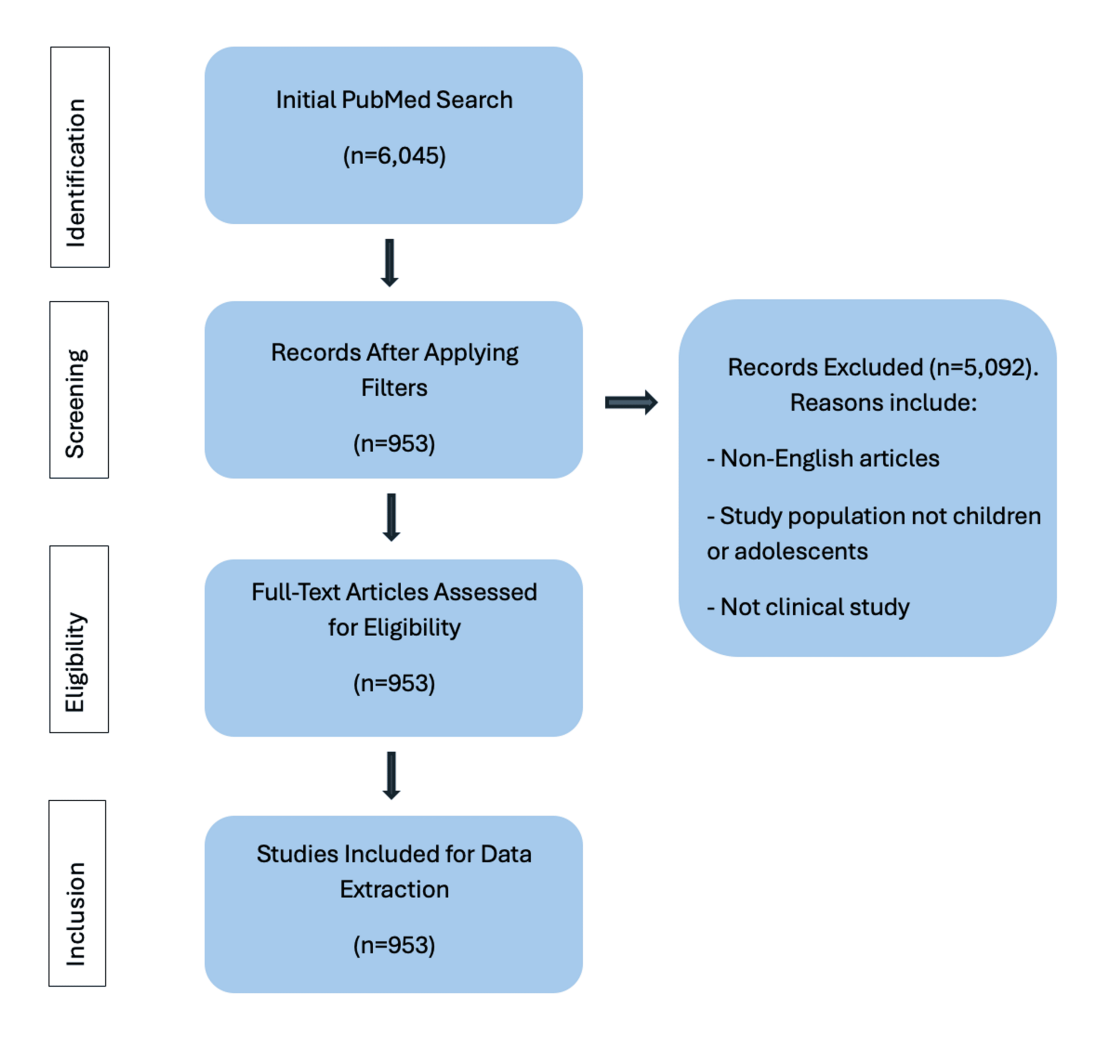
PRISMA Flow Diagram of Study Selection PRISMA: Preferred Reporting Items for Systematic Reviews and Meta-Analyses.

A Preferred Reporting Items for Systematic Reviews and Meta-Analyses (PRISMA) flow diagram summarizing the number of records identified, screened, excluded (with reasons), and included is provided in Figure 1.

Data Extraction

A standardized data abstraction form was developed and piloted using Microsoft Excel (Microsoft, Redmond, WA). For each included article, the following variables were abstracted: (i) *Journal title and type*: Categorized as general scientific, public health, pediatrics, or psychology/behavioral health, based on journal title keywords; (ii) Year of publication; (iii) *Study population*: Classified as children, parents, school nurses, teachers, or coaches. If age was not specified but grade level was provided, age was estimated based on typical U.S. grade-level ages (e.g., first grade=six to seven years old); (iv) *Study design*: Categorized as randomized controlled trial (RCT), observational study, retrospective study, or non-randomized controlled trial. (v) *Research topic(s)*: Derived from keywords in the abstract. Topics included mental health, obesity, physical activity and nutrition, substance use, hygiene, oral health, academic performance, reproductive health, home environment, violence/bullying, and socioeconomic status. Subcategories included: mental health (depression, anxiety, etc.), substance use (alcohol, tobacco, cannabis), and reproductive health (sexually transmitted infections, teen pregnancy); (vi) *Clinical relevance*: Classified as “clinical” if the study included involve an intervention with measurable outcomes; “non-clinical” if the focus was on perceptions, satisfaction, policy, or descriptive analysis; and (vii) *Study outcome*: Defined based on the article’s stated hypothesis and reported as positive, negative, equivocal, or protocol (if describing a planned or in-progress study without reported results).

Note that study population and topic categories were not mutually exclusive. Articles with multiple relevant classifications were tagged under each applicable category.

Data Analysis

Descriptive statistics (total numbers and percentages) were used to summarize all variables. Frequencies and proportions were calculated for categorical data. To assess trends in publication volume over time, a two-sample t-test comparing the number of publications between two time periods (2014-2019 vs. 2020-2024) was performed in consultation with a statistician.

No additional inferential statistics (e.g., meta-analysis, regression modeling) were conducted, as the primary goal was to characterize publication trends and research focus rather than evaluate intervention effectiveness.

Data Visualization and Bibliometric Mapping

To complement descriptive statistics, bibliometric mapping and visualization were conducted using the bibliometrix package in R studio (R Foundation for Statistical Computing, Vienna, Austria) [12]. Although VOSViewer was initially considered, technical constraints related to software compatibility and dependencies limited its function. Bibliometrix was selected as an alternative because it provides comparable, and some cases more flexible, analytical capabilities for bibliometric network visualization, including co-authorship, country collaborations, and keyword co-occurrence analyses.

Two types of bibliometric visualizations were produced: (i) country-specific scientific production, displaying the geographic distribution of publications and highlighting the most prolific contributing countries; and (ii) keyword co-occurrence mapping, illustrating the conceptual structure of the field and revealing frequently co-occurring research terms related to school-based health.

All bibliometric analyses were performed on the final dataset of 953 included publications. Network parameters (e.g., minimum frequency thresholds and normalization methods) were optimized to balance readability and analytical significance.

Risk of Bias and Quality Assessment

A formal risk of bias or quality assessment was not conducted, consistent with the aims and methodology of a bibliometric study. The absence of such analysis is acknowledged as a limitation.

Protocol Registration

This review was not registered in PROSPERO or another protocol database.

Ethics Statement

This study involved secondary analysis of publicly available literature and did not include human subjects or identifiable private information. As such, the study was not submitted for Institutional Review Board (IRB) approval and was not formally reviewed or deemed exempt. No ethical approval was required.

Findings

Publication Trends

A total of 953 publications from 117 journals were analyzed. Of the 953 studies, over 30% were conducted outside of the United States, with representation from regions spanning Asia, Europe, Africa, and South America. Articles appeared mostly in general scientific journals (30%), followed by public health journals (23%), psychology/behavioral health journals (10%), and pediatrics journals (8%). Table 1 reveals the journals with the most publications from our sample and their 2023 impact factors as reported by the Journal Citation Report.

**Table 1:** Top Publishing Journals for Our Sample of School-based Health Studies

Journal Title	Number of Publications	Percentage (%)	2023 Impact Factor
BMC Public Health	149	15.6	3.5
Trials	53	5.6	2.0
BMJ Open	47	4.9	2.9
International Journal of Environmental Research and Public Health	29	3.0	N/A
Public Library of Science One	23	2.4	2.9
International Journal of Behavioral Nutrition and Physical Activity	18	1.9	5.6
Journal of School Health	18	1.9	1.8
Prevention Science	18	1.9	3.0
Contemporary Clinical Trials	16	1.7	2.0
Nutrients	15	1.6	4.8
Journal of Adolescent Health	13	1.4	6.1
Pediatrics	11	1.2	8.0
American Journal of Preventative Medicine	10	1.4	4.3
Implementation Science	10	1.0	8.8
JAMA Pediatrics	10	1.0	26.1

**Figure 2:**
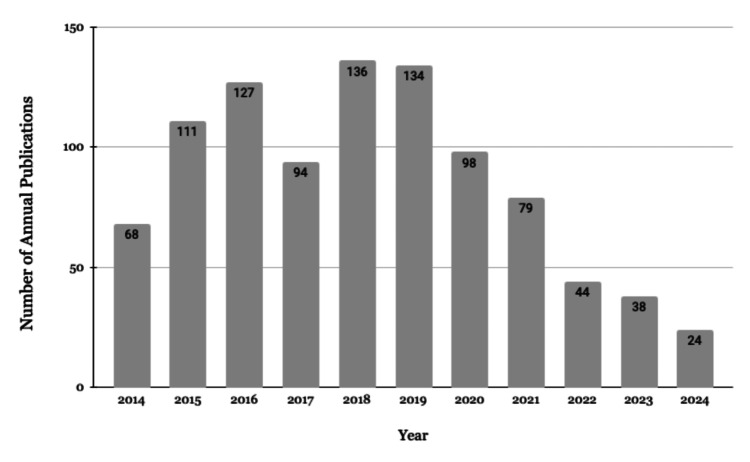
Yearly Trends of School-based Health Publications in Our Sample A peak in annual publications occurred during 2018-2019, followed by a decline in subsequent years. A comparison of publication frequency between the periods 2014-2019 and 2020-2024 revealed a statistically significant decrease (p=0.0111).

Figure 2 illustrates a peak in annual publications during 2018-2019, followed by a decline in subsequent years. A comparative analysis of publication counts between the 2014-2019 and 2020-2024 periods revealed a statistically significant decrease in annual output (p=0.0111), indicating a notable shift in research activity over the past decade.

Country-Specific Scientific Production

**Figure 3:**
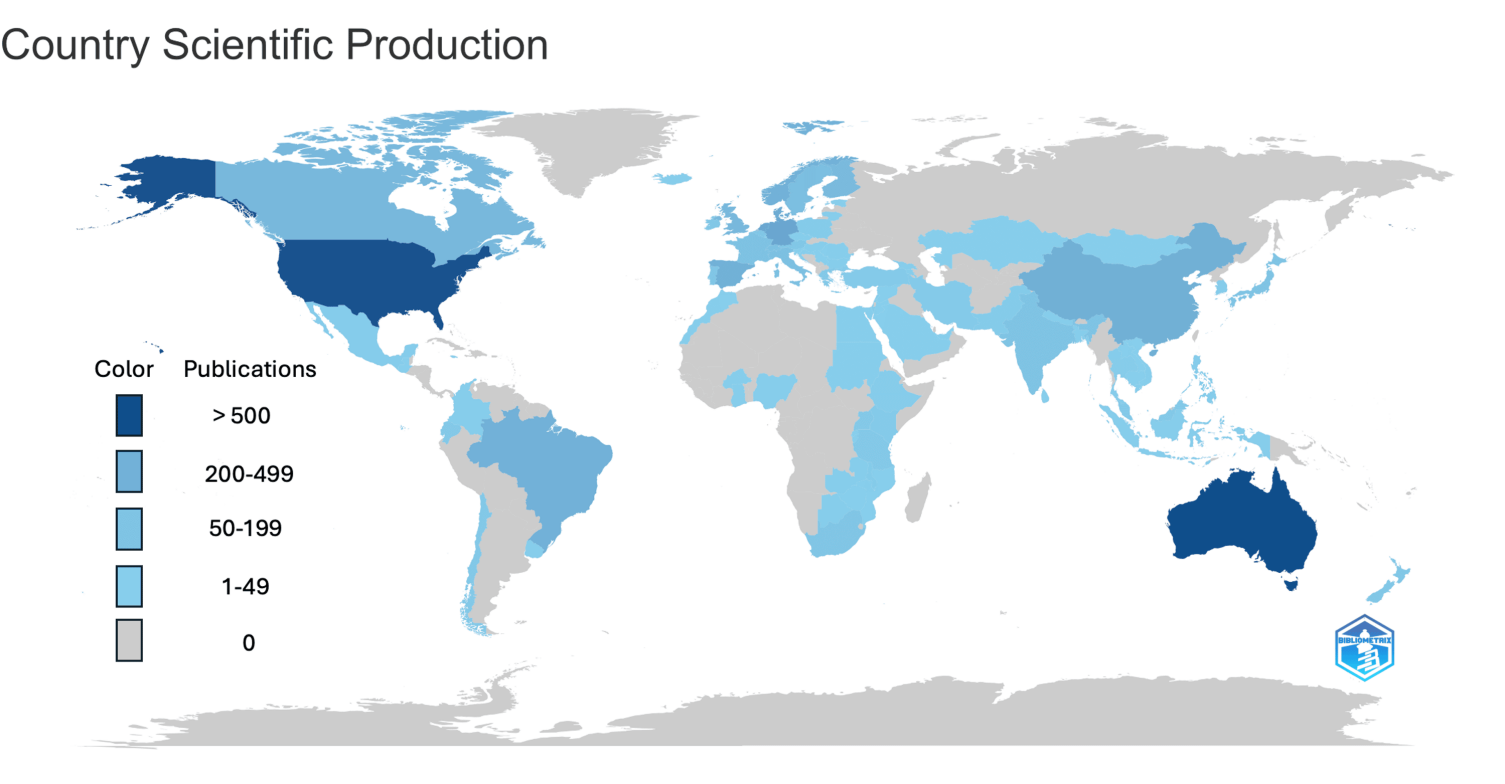
Country-Specific Scientific Production Generated using Bibliometrix in R Studio [12].

Bibliometric mapping revealed a diverse global distribution of school-based health research between 2014 and 2024. Figure 3 presents the global map of country-specific scientific production generated in Bibliometrix. The countries with the highest number of publications were Australia and the United States, followed by Germany, the Netherlands, Denmark, and China. This distribution demonstrates strong engagement from both high-income Western nations and emerging contributors across Asia and South America.

Study Populations

**Figure 4:**
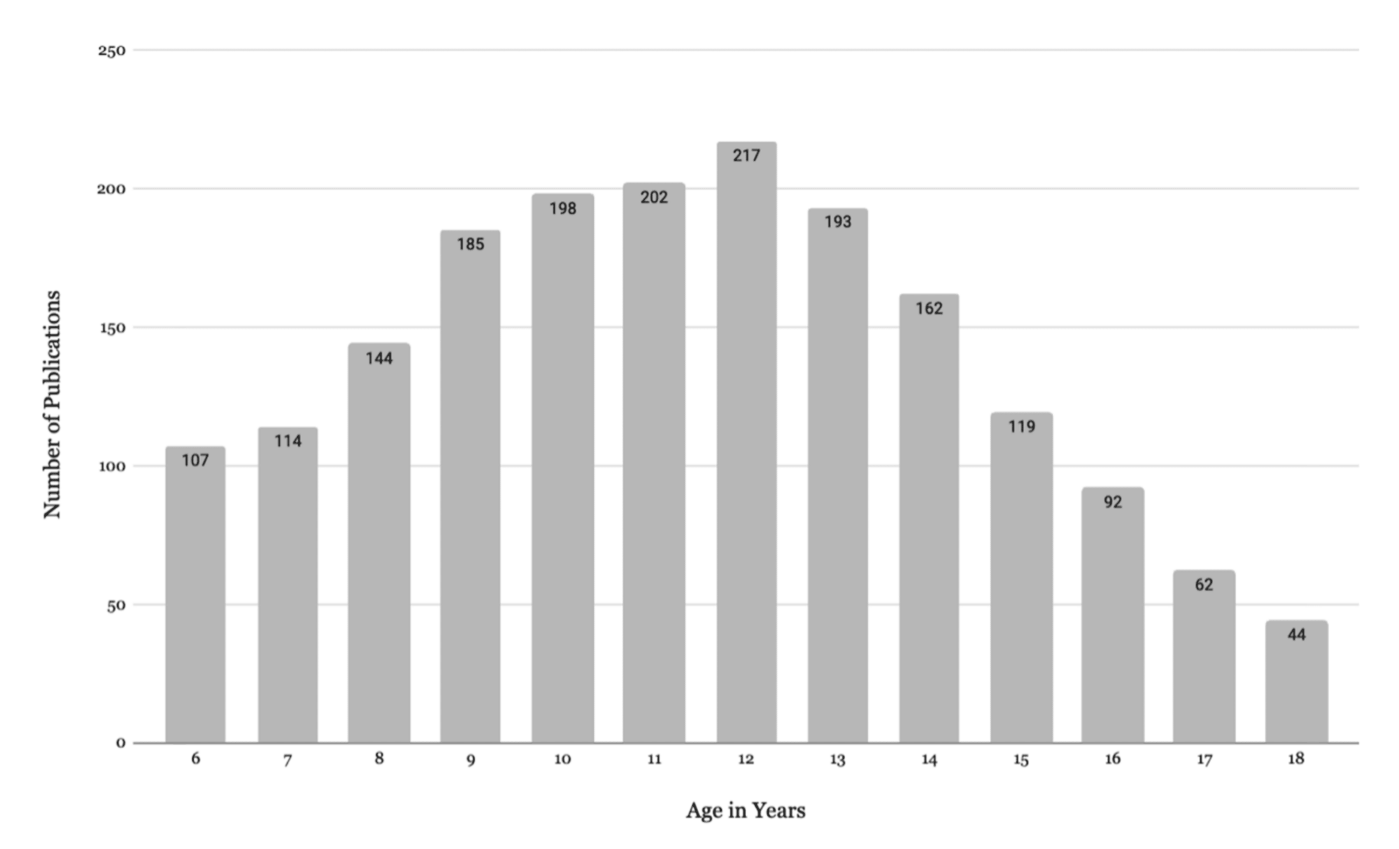
Study Populations for School-based Health Publications in Our Sample

The most frequently studied age group was 12-year-olds, with 51% of publications focusing on children aged nine to 13 years (Figure 4). The figure displays school-aged populations (ages six to 18 years), which were the primary focus of this review. To enhance readability, age groups outside of this range (e.g., early childhood and young adulthood) were excluded from the figure but are included in the analysis.

In total, 79 articles included children aged two to five years; 34 articles included participants aged 19-24 years; 32 articles focused on parents or caregivers; 34 articles studied teachers; three articles included school nurses, and one article examined coaches.

These groups were often included as part of multi-population studies or in the context of intervention delivery or outcome evaluation. While children and adolescents were the predominant focus, the inclusion of educators and caregivers highlights the broader school ecosystem involved in school-based health initiatives.

Study Designs

RCTs were the predominant study design (n=903; 94%), followed by observational studies (5%). Retrospective studies and non-randomized controlled trials each accounted for less than 1% of the studies in our sample.

Keyword Co-Occurrence Network

**Figure 5:**
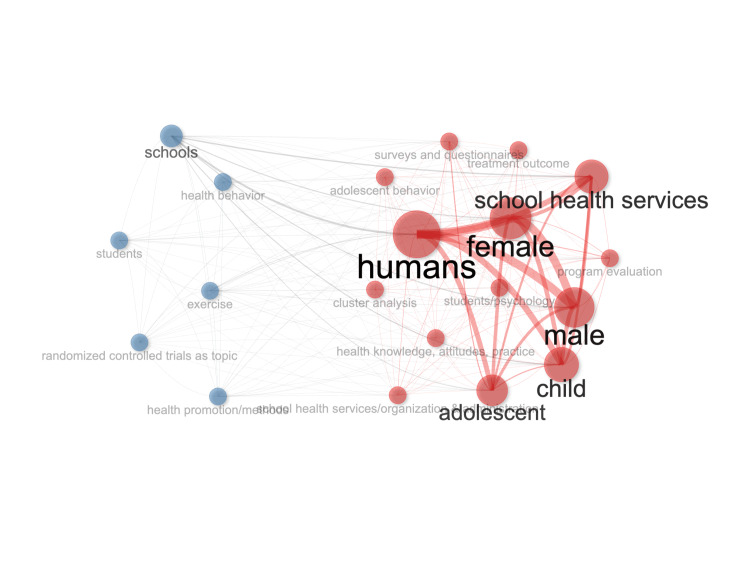
Network Map of Author Keywords Generated using Bibliometrix in R Studio [12].

An analysis of author keywords revealed both broad and topic-specific trends. Figure 5 displays the keyword co-occurrence network generated using the Bibliometrix package in R. The most frequently occurring general keywords were humans, female, male, child, adolescent, and school health services. Beyond these, more specific research topics were highlighted, including health behavior, exercise, health promotion, health knowledge and attitudes, and adolescent behavior. These keywords suggest the primary focus areas of the literature in the dataset.

Research Topics

Table 2 summarizes research study topics covered in the publications. The most commonly assigned research topics were physical activity and nutrition (36%), health promotion/education (25%), and mental health (19%).

**Table 2:** Top Research Topics in Our Sample of School-based Health Studies* *Does not equal 953 because some articles had more than one research study topic assigned to them.

Research Study Topic	Number of Articles	Research Study Topic	Number of Articles
Physical activity and nutrition	344	Academic performance	42
Physical activity	161	Wellness/Mindfulness	40
Nutrition	74	Oral Health	32
Health promotion/education	236	Hygiene	27
Mental health	176	Risk behavior	26
Depression	34	Asthma and allergies	17
Cognitive behavioral therapy	31	Vaccinations	14
Body image/eating disorders	25	Screen time and video games	12
Anxiety	10	Program evaluation	12
Suicide	10	Cardiovascular	11
Autism	10	Safety	9
Attention-deficit/hyperactivity disorder	9	Sports medicine	9
Post-traumatic stress disorder	2	Concussions	3
Obesity	145	Injuries	3
Substance use	117	Physical therapy	3
Tobacco	46	Helminth infections	8
Alcohol	37	Intellectual disability	7
Cannabis	16	Sun protection	7
Socioeconomic status	64	Type 2 Diabetes Mellitus	6
Violence and bullying	58	Afterschool programs	6
Violence	34	Influenza	6
Bullying	16	Sleep health	6
Abuse	8	Cancer	5
Home environment and parents	50	Recess	5
Home environment	32	COVID-19	5
Parents	18	Vision	4
Reproductive Health	45	Malaria	3
* *General health promotion	23	Cardiopulmonary Resuscitation	3
* *Human immunodeficiency virus	8	Organ donation	2
* *Teen pregnancy	7	Anemia	2
* *Sexually transmitted infections	4	Telemedicine	2
* *Human papilloma virus	3	Hydration	1

Clinical Focus and Research Outcomes

Clinically focused publications comprised the majority of relevance in our sample (97%), with 3% considered non-clinical studies. Regarding research outcomes, 60% reported positive findings, 17% equivocal, less than 1% negative, and 22% were protocols.

Discussion

This bibliometric review reveals a robust and diverse body of literature on school-based health published between 2014 and 2024. The inclusion of 953 studies across a wide array of journals, including high-impact publications, underscores the field’s growing relevance and interdisciplinary importance. The dominance of clinical publications and RCTs reflects a strong evidence-based foundation for school-based interventions, yet also signals potential blind spots in the literature that merit further exploration.

Physical Activity and Nutrition

The most frequent topic among the publications was physical activity and nutrition, a predictable yet concerning finding given the rising rates of childhood obesity worldwide. 

Interventions in our sample focused on incorporating physical activity and nutrition education into the curriculum and improving access to healthy foods. These efforts align with global public health goals, as reducing body mass index is linked to improved long-term outcomes, including lower risk of diabetes and cardiovascular disease [13]. The persistent focus on this area likely reflects both its global urgency and the relative ease of measuring outcomes such as weight, physical activity levels, and dietary intake in school settings.

Health Promotion and Education

A substantial portion of studies focused on health education, particularly relating to hygiene, sun protection, and substance use prevention. These interventions are vital in shaping lifelong behaviors, especially given global deficits in health literacy that were magnified during the COVID-19 pandemic. Promoting students’ health at school has been found to encourage healthy habits and increase health literacy and has also engaged families and communities, thus impacting the entire society [14]. This is essential, as family involvement will further promote healthy behaviors, and consistent encouragement from all aspects of a child’s life leads to better outcomes.

Mental Health

Mental health emerged as a prominent topic, reflecting the global rise in youth mental health disorders, now estimated to affect nearly 15% of children and adolescents [15]. Depression, anxiety, and eating disorders were frequently studied. The growing attention to youth mental health is likely fueled by increased awareness, reduced stigma, and enhanced policy prioritization. Given that 50% of mental health disorders emerge by age 14, and 75% by the age of 24 [16], mental health in our youth population has become a universal concern and addressing it has become a global priority.

Substance Use

Tobacco and alcohol were the primary substance-related topics covered, aligning with evidence that early initiation leads to a higher risk of dependence and long-term harm, including premature death [17-19]. This ongoing focus suggests recognition of schools as a critical venue for early intervention. However, newer substances such as vaping and cannabis, which are increasingly popular among adolescents, appear underrepresented in the literature and warrant greater attention.

Under-Researched Areas and Gaps

Although issues like socioeconomic status, violence, reproductive health, and intellectual disability were present, they were comparatively underrepresented.

Socioeconomic Status

It is increasingly important that future studies expand on the impact that school-based health interventions can have on children and adolescents with low socioeconomic status. In addition to the barriers that these youth face with access to health care [7], they are also challenged by an unhealthy lifestyle due to poor availability of goods and resources. Youth with low socioeconomic status have been shown to have an increased risk of indulging in unhealthy behaviors, such as earlier initiation of tobacco use, increased consumption of unhealthy foods, lower levels of physical activity, and more involvement in drug abuse [20]. Continuing to introduce school-based health interventions will enhance the health behaviors and outcomes of children and adolescents, having a particularly profound effect on those from a low socioeconomic background.

Violence

The effect that violence has on youth in our society is growing, and preventing youth violence should be a priority in schools and communities. Youth violence leads to increases in deaths, injuries, disability, mental health problems, and even chronic diseases, as well as its impact on development and social functioning [21]. Bullying has been shown to lead to increased rates of substance use, mental health issues, and suicide, and some even retaliate with violence themselves. In the 1990s, 12 out of 15 school shooters reported being bullied [22]. Youth violence can be addressed by initiating social development programs focused on anger management, identifying and offering therapy to students who are identified as high risk for engaging in violent behavior, and incorporating whole-school violence prevention education [21].

Reproductive Health

Many youth face barriers to sexual and reproductive health education and resources because of which they practice and establish unsafe health behaviors. In our sample of publications, the most common research topics focused on teen pregnancy and sexually transmitted infections, particularly HIV and human papillomavirus (HPV). Between 2011 and 2021, condom use in adolescents has decreased by 8%, and while this decline is likely associated with the increase in hormonal forms of contraception, condoms are still the only method of birth control that prevents sexually transmitted infections (STIs) [23]. The incidence of STIs is increasing in adolescents, resulting in adolescents making up 50% of diagnosed STIs every year [24]. Though the overall rate of adolescent pregnancy has declined, recent data demonstrates variation between regions, education levels, and socioeconomic statuses. The factors that contribute to the variation includes child marriage, limited reproductive health education, and poor access to contraceptives [25]. Thus, there is potential for future school-based research and interventions to focus on and address these issues.

Intellectual Disability

A population with an increased need for school-based interventions and research includes students with intellectual disability. An estimated 1%-3% of the global population, about 200 million people, live with intellectual disability [26]. People with intellectual disability have an increased risk of cardiovascular disease, obesity, and age-related diseases. However, their reduced ability to communicate and understand leads to a lack of health knowledge and difficulties with health promotion and behavior [27], and therefore they are a particularly vulnerable group. As more children with disabilities are beginning to attend school, school becomes an even more essential platform to reach these children and adolescents.

Telemedicine

An increasingly growing area for research is telemedicine and the benefits for patients and providers. Although telemedicine surged following the COVID-19 pandemic [28], very few articles in our sample examined its use in school-based settings. This omission is surprising given its potential to address access barriers, particularly in rural or underserved communities.

School-Based Health Centers

The underrepresentation of SBHCs in the literature is especially notable. Despite serving over two million children in the United States, and the benefit of increased learning opportunities, help-seeking behaviors, and trust in their doctor [29], no studies in our sample directly examined their impact. Because of the positive outcomes and the growth potential seen with SBHCs, this remains a topic for future studies and an area for growth in research.

Trends in Publication Volume

Our data show a statistically significant decline in school-based health publications after 2019. While the COVID-19 pandemic likely played a major role, disrupting research activities, diverting funding, and closing schools, other factors may also contribute [30]. These include shifting policy priorities, changes in global funding streams, or database indexing delays. Future studies should examine whether this decline persists post-pandemic or reflects a temporary disruption.

Limitations

This study has several limitations that may affect generalizability and interpretation.

*Database selection*: The search was conducted using only PubMed, a database with strong coverage of biomedical literature. Databases such as SCOPUS may contain additional relevant publications. Including multiple databases would provide a more comprehensive view of the interdisciplinary nature of school-based health.

*Language and geographic bias*: The search was limited to English-language articles, which could bias the results toward English-speaking or English-publishing countries. Although the articles were all in English, over 30% of the 953 studies were conducted outside the United States, reflecting a degree of international scope. However, this language restriction still limits the generalizability of our findings to non-English-speaking regions. Future research should incorporate non-English databases to capture a broader global perspective.

*Publication types*: Our analysis focused exclusively on clinical studies and excluded case reports, reviews, commentaries, and policy briefs. While this decision ensured consistency in study type, it may underrepresent important insights from qualitative, observational, or policy-driven research. Moreover, the predominance of RCTs may limit understanding of broader contextual factors affecting intervention outcomes.

*Timeframe*: The analysis includes articles published between January 2014 and July 2024. Studies published mid-2024 to present were not included and could impact observed trends. Future updates should expand the date range to include these articles.

*Reviewer bias*: All stages of screening, full-text review, inclusion/exclusion decisions, and data extraction were conducted by a single reviewer. While this approach ensured consistency in decision-making, it also increases the risk of subjective bias, misclassification, or unintentional exclusion of relevant studies. The absence of a second reviewer may limit the reliability of screening decisions and the reproducibility of the analysis. Future bibliometric reviews would benefit from involving multiple reviewers and inter-rater reliability checks to improve methodological rigor.

*No quality assessment*: While we analyzed publication trends, study designs, and topics, we did not assess the methodological quality, rigor, or impact of each study. Therefore, conclusions are based on volume and scope rather than evidence strength or outcomes.

*Publication bias*: Our finding that most studies reported positive outcomes should be interpreted with caution, as studies with favorable results are more likely to be published. This potential for publication bias may overestimate the perceived effectiveness of school-based interventions. Future analyses should consider including unpublished or gray literature to mitigate this limitation.

Future directions

To build a more equitable and effective body of school-based health research, future studies should focus on broadening the scope of inquiry and strengthening methodological diversity. Particular attention should be given to underrepresented populations, including immigrant youth, LGBTQ+ (lesbian, gay, bisexual, transgender, queer) students, and students with disabilities, whose unique health needs remain insufficiently studied. Expanding research on school-based health centers and evaluating their impact through both clinical and policy-oriented approaches could provide valuable insights into their effectiveness and scalability. Similarly, the growing role of telehealth in schools warrants rigorous evaluation to determine its long-term feasibility, accessibility, and outcomes, particularly in rural and underserved areas.

Future work should also address geographic and linguistic gaps by including studies published in non-English languages to capture a more global perspective. Moreover, complementing traditional randomized controlled trials with mixed-methods and community-based research designs could yield a more comprehensive understanding of contextual factors that influence implementation and success. By diversifying research populations, settings, and methodologies, scholars can help ensure that school-based health systems evolve in ways that are inclusive, sustainable, and evidence-informed.

## Conclusions

This bibliometric review highlights key trends in school-based health research from 2014 to 2024, demonstrate both the field’s growth and the challenges it currently faces. Although the volume of publications increased early in the decade, a decline after 2019 raises questions about the sustainability of research activity and potential shifts in global priorities. Despite its reduction, most studies reported positive outcomes, reinforcing the value of school-based interventions for improving the health and well-being of children and adolescents. Nevertheless, the predominance of favorable results may reflect publication bias and should be interpreted with caution.

Moving forward, it will be crucial for researchers to address ongoing gaps in the literature, particularly those related to school-based health centers, telemedicine, and the health needs of underserved groups such as immigrant, disabled, and LGBTQ+ students. Additional focus on reproductive health, violence prevention, and socioeconomic disparities will further advance the field. Expanding research in these areas and adopting more diverse study designs will strengthen the evidence base and ensure that school-based health programs equitably serve all students. By capturing evolving trends and spotlighting critical areas for growth, this study underscores the importance of continued investment in school-based health research to promote the well-being of the global pediatric population.
